# Iron Overload Impairs Bone Marrow Mesenchymal Stromal Cells from Higher-Risk MDS Patients by Regulating the ROS-Related Wnt/*β*-Catenin Pathway

**DOI:** 10.1155/2020/8855038

**Published:** 2020-10-31

**Authors:** Lei Huang, Zhaoyun Liu, Hui Liu, Kai Ding, Fu Mi, Chenhuan Xiang, Guanrou Wang, Yixuan Guo, Rong Fu

**Affiliations:** Department of Hematology, Tianjin Medical University General Hospital, Tianjin 300052, China

## Abstract

The bone marrow microenvironment plays important roles in the progression of the myelodysplastic syndrome (MDS). The higher incidence of ASXL1 and TET2 gene mutations in our iron overload (IO) MDS patients suggests that IO may be involved in the pathogenesis of MDS. The effects of IO damaging bone marrow mesenchymal stromal cells (MSCs) from higher-risk MDS patients were investigated. In our study, IO decreased the quantity and weakened the abilities of proliferation and differentiation of MSCs, and it inhibited the gene expressions of VEGFA, CXCL12, and TGF-*β*1 in MSCs regulating hematopoiesis. The increased level of reactive oxygen species (ROS) in MSCs caused by IO might be inducing apoptosis by activating caspase3 signals and involving in MDS progression by activating *β*-catenin signals. The damages of MSCs caused by IO could be partially reversed by an antioxidant or an iron chelator. Furthermore, the MSCs in IO MDS/AML patients had increased levels of ROS and apoptosis, and the expressions of caspase3 and *β*-catenin were increased even further. In conclusion, IO affects gene stability in higher-risk MDS patients and impairs MSCs by inducing ROS-related apoptosis and activating the Wnt/*β*-catenin signaling pathway, which could be partially reversed by an antioxidant or an iron chelator.

## 1. Introduction

The myelodysplastic syndrome is a clonal stem-cell-derived myeloid neoplasm, which is characterized by ineffective hematopoiesis, presenting with decreased hematopoiesis in one or more blood cells, varied proliferation degrees of blast cells, and a high-risk transformation to acute myeloid leukemia (AML) [1]. Abnormal hematopoietic stem cells (HSCs), bone marrow stromal cells, and immune cells are related to the occurrence and development of MDS. Meanwhile, HSCs with gene mutations gain the advantage of proliferation and develop into malignant cells [2].

The bone marrow microenvironment is important in regulating the quantity, functions, and renewal of HSCs. The functions of MSCs are abnormal in MDS patients, with decreased abilities in supporting hematopoiesis [3]. Similarly, the quantity of HSCs in MDS mice (NHD13 mice) decreased, with increased vascular endothelial cells and reduced quantity and functions of MSCs, which were involved in the progression of MDS mice [4]. Previous studies indicated that MSCs played an important role in hematopoiesis, and in turn, abnormal HSCs might affect the functions of MSCs in MDS patients. Meanwhile, abnormal MSCs might facilitate the proliferation of malignant cells, affect the expressions of hematopoietic cytokines, influence immune cells by secreting varieties of inflammatory cytokines, and promote MDS transformation to AML (MDS/AML) [5]. Therefore, factors affecting the bone marrow microenvironment may be involved in the progression of MDS.

IO is very common in MDS patients: in most patients, IO is caused by chronic blood transfusions, while in others, it is caused by ineffective hematopoiesis or abnormal regulation of hepcidin [6–8]. IO affected the survival of MDS patients, and serum ferritin exceeding 1000 ng/ml often indicated poor prognosis, while iron chelation therapy might prolong the survival time of MDS patients [9–11]. Previous studies showed that the effects of IO on MSCs are caused partly via inducing mitochondrial injury and ROS-related apoptosis [12, 13], but the other mechanisms involved are yet undetermined. The Wnt/*β*-catenin signaling pathway played an important role in the occurrence and development of MDS. Wnt-related proteins participate in normal hematopoiesis, but its dysregulation is involved in the progression of various kinds of leukemia. In the absence of Wnt signals, *β*-catenin in the cytoplasm was maintained at a very low level. Ser/Thru kinase (GSK-3*β*) and casein kinase 1 (CK1) phosphorylated the conserved regions of *β*-catenin and deactivated it by proteasomal degradation [14]. Wnt signals were abnormal in the MSCs of MDS patients, and the defects of MSCs might be related to the occurrence and progression of MDS [15]. However, it is unclear whether iron overload may be involved in the progression of MDS by affecting Wnt-related signaling pathways.

In our study, we try to determine the effects of IO on the quantity and functions of MSCs, and the mechanisms involved in the damages of MSCs caused by IO in patients with intermediate or high-risk MDS. Moreover, the roles of IO in the progression of MDS are also investigated in order to provide more evidences for iron chelation therapy in MDS patients.

## 2. Patients and Methods

### 2.1. Patients and Healthy Volunteers

From October 2017 to December 2019, forty higher-risk MDS patients, thirteen IO MDS/AML patients diagnosed in the Department of Hematology, Tianjin Medical University General Hospital, and nineteen healthy volunteers were recruited in our study, including 23 males and 30 females. The median age of the patients was 61 years (range 26-85). The median levels of ferritin were 427.28 (32.72 ~ 874.57) ng/ml in the NIO MDS group, 2000 (1012.85 ~ >2000) ng/ml in the IO MDS group, and 1831.1 (1040.8 ~ >2000) ng/ml in MDS/AML patients. The diagnosis and classification of MDS were performed according to the 2016 World Health Organization (WHO) and Revised International Prognostic Scoring System (IPSS-R) recommendations [16]. Nine MDS patients progressed to AML after demethylation therapy, and the other four patients with dysplastic features had a history of MDS without receiving demethylation treatment before transformation. IO was defined as serum ferritin above 1000 ng/ml. No obvious difference was found in the distribution of gender, WHO classification, and IPSS-R between the IO MDS groups and the NIO MDS groups (*p* > 0.05) (Table 1).

### 2.2. Gene Mutation Analysis

The bone marrow mononuclear cells (BMMNC) from MDS patients were isolated by centrifugation in a Ficoll gradient. Intracellular DNA was extracted for subsequent sequencing tests. Next-generation sequencing (NGS) was used to screen the gene mutations in BMMNC from the higher-risk MDS patients, sequencing by Ion Torrent PGM sequencer/Illumina sequencer. Transcript ID was also extracted. A transcript is a transcription of one or more mature mRNAs that can be encoded by a gene. According to NCBI database, different splicing of introns on a piece of gene can form different transcription IDs, or transcription number. Mutation site refers to the exon region or protein functional domain where the mutation site is located on the transcript. The list of gene mutations is presented in the supplementary material (Table S1).

### 2.3. Sample Collection and Cell Culture

Heparin anticoagulant aseptic bone marrow 5 ml was extracted from patients and volunteers and centrifuged at 1500 revs/min for 10 min. The bone marrow supernatant was stored in a 1.5 ml EP tube and stored in a -80°C refrigerator for later use. The BMMNC from patients and volunteers were isolated by centrifugation in a Ficoll gradient. 2 × 10^5^/ml cells were inoculated in six-well plates and cultured with DMEM/F12 (Gibco) supplemented with 15% fetal bovine serum (FBS, Gibco), 100 mg/ml penicillin (Gibco), and 100 U/ml streptomycin (Gibco) at 37°C in a 5% CO_2_ atmosphere. After 72 h, half of the culture medium was changed, and then the culture medium was changed once every 3-4 days. After about two weeks, the culture medium was discarded when the adherent cells grew to 80-90% fusion. 0.25% trypsin was used to digest the adherent cells, then the cells were resuspended by adding DMEM/F12 culture medium and passed on at a ratio of 1 : 2. The third passage MSCs were digested by 0.25% trypsin, resuspended by adding DMEM/F12 culture medium, and used in the following experiments. To the MSCs from the IO MDS group, cells were divided into three groups: one grouped was used as a positive control, the other group was cultured with 100 *μ*M deferoxamine (DFO) (TargetMol) for 24 h, and the last group was cultured with 5 mM N-acetylcysteine (NAC) (Beyotime Biotechnology, China) for 24 h.

### 2.4. The Purity of MSCs

The third passage MSCs were collected and washed twice with PBS. Then, the cells were incubated with mouse anti-human CD34-PerCP, CD45-APC-CY7, CD105-APC, CD73-PE, and CD90-FITC for 20 min at room temperature in the dark. Finally, the cells were washed once with PBS, 200 *μ*l PBS was added, and the solution was analyzed by a flow cytometer (Beckman CytoFLEX). CytExpert2.3 software platform was used to analyze the data.

### 2.5. Iron Staining

MSCs were seeded at a density of 1 × 10^5^/well in 6-well plates in growth medium. An iron-staining fixative was added to each well for 10 min at room temperature, and then the wells were washed with PBS and dried. Liquid iron stain assays were added to each well incubating in a 37°C water bath for 1 h, washed and redyed with nuclear solid red for 2 min, and then washed and dried and observed in an inverted microscope. Positive results were indicated by the presence of blue deposits in the cells.

### 2.6. Cell Viability Assays by CCK8

The proliferation of MSCs from 15 healthy controls, 18 NIO MDS patients, and IO MDS patients was examined. MSCs were seeded at a density of 2 × 10^3^/well in 96-well plates in growth medium. Cells were cultured by replacing growth medium every two days. Every other day, we took out a 96-well plate to detect the cell viability till twelve days. 10 *μ*l CCK8 (Engreen, China) was added to each well and incubated for another 3 h at 37°C in a 5% CO_2_ atmosphere. The absorbance of the samples was measured against a background control at 450 nm. The experiment was repeated 3 times, and each sample had 3 duplications. Cell viability was calculated using the following equation: proliferation (%) = (OD_450_ of the experimental group/OD_450_ of the control group) × 100%.

### 2.7. Osteogenic Induction of MSCs

For osteogenic induction, cells were plated at a density of 5 × 10^4^/well in a 24-well plate cultured in DMEM/F12 supplemented with 15% FBS, 100 mg/ml penicillin, 100 U/ml streptomycin, 10^−7^ M dexamethasone, 0.2 mM vitamin C, and 10 mM glycerin-phosphate at 37°C in a 5% CO_2_ atmosphere. After 3 weeks of induction, cells were washed with PBS, fixed with 10% paraformaldehyde, and then washed and stained with silver nitrate (Sigma-Aldrich, USA) for 30 min in order to observe phosphate mineralization in the extracellular matrix as a late marker of osteogenesis; calcium nodules were observed under an inverted microscope.

### 2.8. qRT-PCR Analysis

Total RNA was extracted from cells using the TRIzol Reagent (Tiangen Biotech, China) according to the manufacturer's recommendations. The first-strand cDNA was synthesized from 1 *μ*g of total RNA using the FastQuant RT Kit (with gDNase) (Tiangen Biotech, China). Subsequently, a quantitative polymerase chain reaction (qPCR) was performed in triplicate on the IQ5 (Bio-Rad, USA) with SuperReal PreMix Plus SYBR Green (Tiangen Biotech, China) and primers (10 *μ*M), and the amplified specific single product was validated by a melt curve. The following gene-specific primers are shown in Table 2.

### 2.9. Detection of TGF-*β*1 in the Culture Supernatant by ELISA

The culture supernatants secreted by MSCs were collected. The levels of TGF-*β*1 were measured using the Human TGF-beta1 ELISA Kit according to the manufacturer's instructions (Multisciences, China).

### 2.10. ROS Detection by Flow Cytometry

The generation of intracellular ROS was determined using a fluorescein-labeled dye, 2′,7′-dichlorofluorescein diacetate (DCFH-DA), (Beyotime Biotechnology, China) following the manufacturer's protocol. MSCs were collected and washed once with PBS. Then, the cells were incubated with 10 *μ*M DCFH-DA in a 5% CO_2_ atmosphere at 37°C for 20 min. Finally, cells were washed and analyzed by a flow cytometer (Beckman CytoFLEX). The CytExpert 2.3 software platform was used to analyze the data.

### 2.11. Cell Apoptosis Assays by Flow Cytometry

MSCs were harvested and washed with apoptosis-binding buffer and resuspended in 200 *μ*l staining solution containing 5 *μ*l Annexin V-FITC and 5 *μ*l PI-ECD (FITC-Annexin V staining kit, BD) at room temperature in the dark for 30 min. Then, cells were detected by a flow cytometer (Beckman CytoFLEX), and the percentage of apoptosis in the total number of cells in each group was compared. The CytExpert 2.3 software platform was used to analyze the data.

### 2.12. Western Blot Analysis

Total cell lysates were extracted using the RIPA lysis buffer containing PMSF (100 : 1), and the protein concentrations were determined with the BCA protein assay kit (Sangon Biotech, China). Proteins (30 *μ*g) were separated by 10% SDS-PAGEs and transferred electronically to NC membranes (EASYBIO, China). NC membranes were blocked with 5% BSA for 1 h at room temperature (RT), and then incubated overnight at 4°C with the following primary antibodies: anti-AKT (#4691S), anti-p-AKT (#9271S), anti-GSK-3*β* (#12456S), anti-p-GSK-3*β* (#5558S), anti-*β*-catenin (#8480T), anti-caspase3 (#9668S), and anti-GAPDH (#5174T) (Cell Signaling Technology, USA). After washing three times with TBST, each membrane was incubated with anti-mouse IgG (GenScript A00160) or anti-rabbit IgG (GenScript A00098) as a secondary antibody for 1 h at room temperature. The expressions of the proteins of interest were examined by enhanced chemiluminescence kits (Millipore, Billerica, MA, USA).

### 2.13. Statistical Analysis

The results are presented as mean ± standard deviation or median (minimum, maximum). Data with normal distribution and homogeneity of variance were analyzed using independent Student's *t*-test or ANOVA. Abnormal distribution of two-paired samples were compared by nonparametric analysis with Wilcoxon's test, while comparisons of enumeration data were compared by Pearson's chi-square, continuity correction, or Fisher's exact test. All statistical analyses were done using the statistical software SPSS version 21.0. *p* value of <0.05 was considered statistically significant.

## 3. Results

### 3.1. Gene Mutations between the IO and NIO MDS Groups

Due to the effects of chromosomal instability in the progression of MDS, we analyzed the gene mutations in higher-risk MDS patients. NGS was performed to detect 34 gene mutations related to the pathogenesis of MDS, and we found that 10/10 IO MDS patients and 8/11 NIO MDS patients had gene mutations, while 3/11 NIO MDS patients were negative in the gene mutation test. Further analysis showed that among the 34 gene mutations, the incidence of ASXL1 and TET2 gene mutations was significantly higher in the IO MDS group (6/10, 7/10) than that in the NIO MDS group (1/11, 2/11) (*p* < 0.05) (Table 3), which suggested that the BMMNC from IO MDS patients might be more prone to genetic abnormalities and damaged by iron overload.

### 3.2. The Characteristics and Iron Staining of MSCs

**Figure 1:**
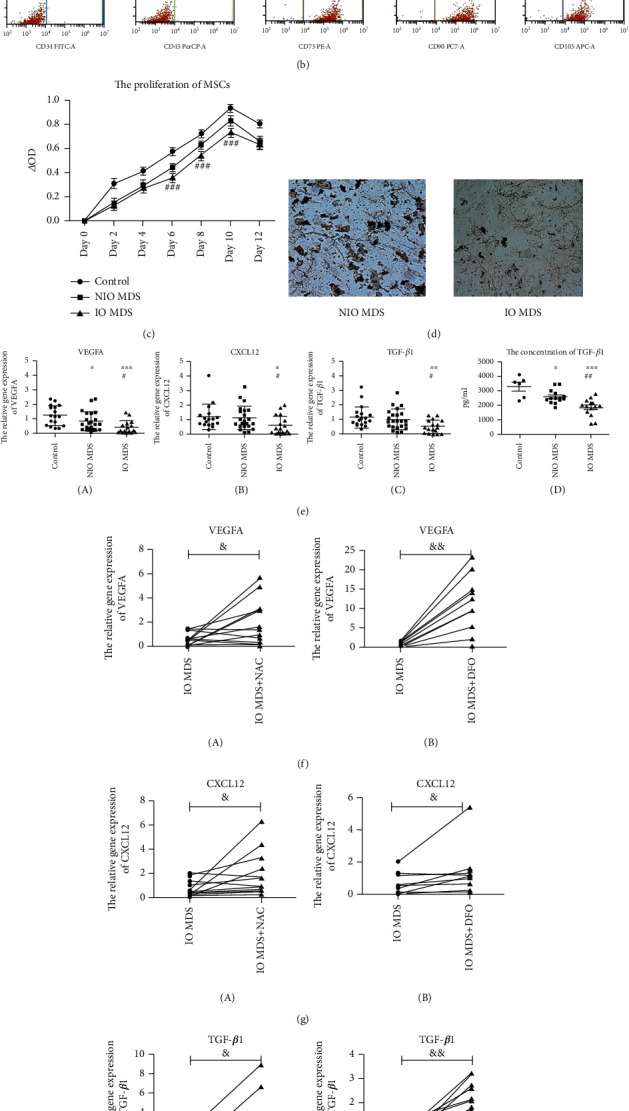
The characteristics and functions of MSCs isolated from intermediate or high-risk MDS patients. (a-A) The IO MDS group had a decreased quantity of MSCs without morphological differences. (a-B) The iron deposition in the IO MDS group was increased. (b) The purity of MSCs was over 90% as examined by FCM. (c) The proliferation ability of MSCs from MDS patients was significantly decreased compared with that of the controls; furthermore, the proliferation ability of MSCs in the IO MDS group was significantly decreased compared with that in the NIO MDS group on days 6, 8, and 10 (*p* < 0.05). (d) The osteogenic differentiation ability of MSCs in the IO MDS group was lower than that in the NIO MDS group. (e) The gene expressions of VEGFA, CXCL12, and TGF-*β*1 regulating hematopoiesis were significantly downregulated in the IO MDS group, as well as the level of TGF-*β*1 in the culture supernatants secreted by MSCs. (f–h) The gene expressions of VEGFA, CXCL12, and TGF-*β*1 in IO MDS MSCs were significantly upregulated after the treatments with NAC or DFO (*p* < 0.05). Compared with the control group: ∗ indicates *p* < 0.05; ∗∗ indicates *p* < 0.01; ∗∗∗ indicates *p* < 0.001. Compared with the NIO MDS group: # indicates *p* < 0.05; ## indicates *p* < 0.01; ### indicates *p* < 0.001. Compared with the IO MDS group: & indicates *p* < 0.05; && indicates *p* < 0.01.

MSCs were isolated and induced by BMMNC in vitro. Compared with the NIO MDS and control groups, the IO MDS group had a decreased quantity of MSCs without obvious morphological differences (Figure 1(a) A). The iron deposition of MSCs increased in the IO MDS group compared with that in the NIO MDS and control groups (Figure 1(a) B). The purity of MSCs was over 90% examined by FCM (Figure 1(b)).

### 3.3. IO Inhibited the Proliferation and Differentiation Abilities of MSCs

**Figure 2:**
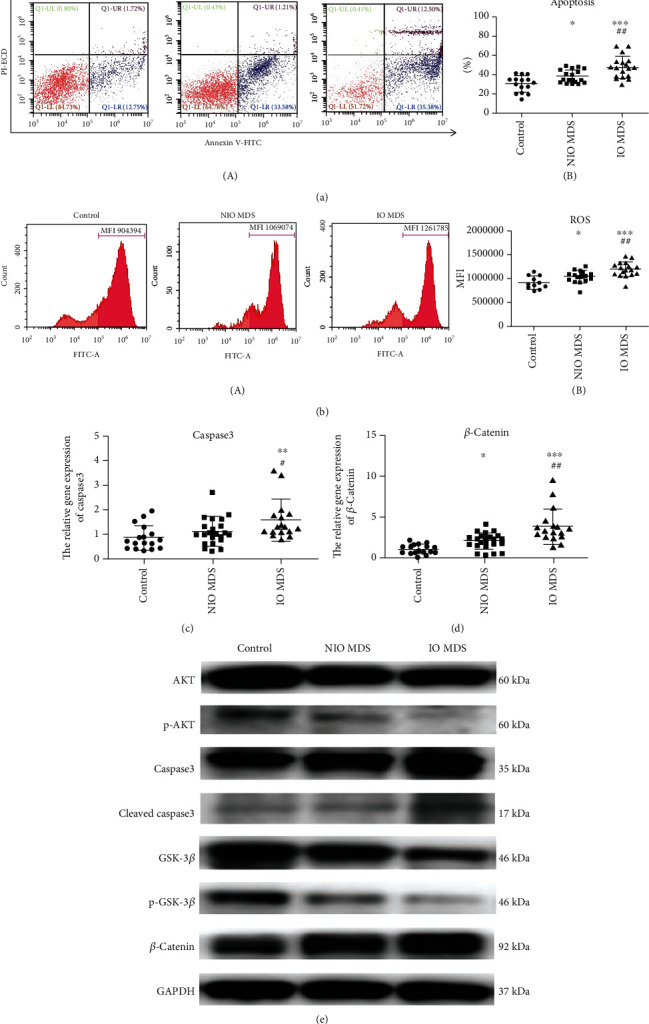
The mechanisms involved in IO damaging MSCs of MDS patients. (a) Compared with the NIO MDS group and the control group, the apoptosis of MSCs significantly increased in the IO MDS group (*p* < 0.01). (b) Compared with the other groups, the level of ROS significantly increased in MSCs of the IO MDS group (*p* < 0.01). (c–d) The gene expressions of caspase3 and *β*-catenin were significantly upregulated in MSCs of the IO MDS group compared with those of the other groups. (e) The protein of p-AKT in IO MDS MSCs was downregulated, while caspase3 was upregulated. The protein of *β*-catenin in IO MDS MSCs was upregulated, while GSK-3*β* was downregulated. Compared with the control group: ∗ indicates *p* < 0.05; ∗∗ indicates *p* < 0.01; ∗∗∗ indicates *p* < 0.001. Compared with the NIO MDS group: # indicates *p* < 0.05; ## indicates *p* < 0.01.

**Figure 3:**
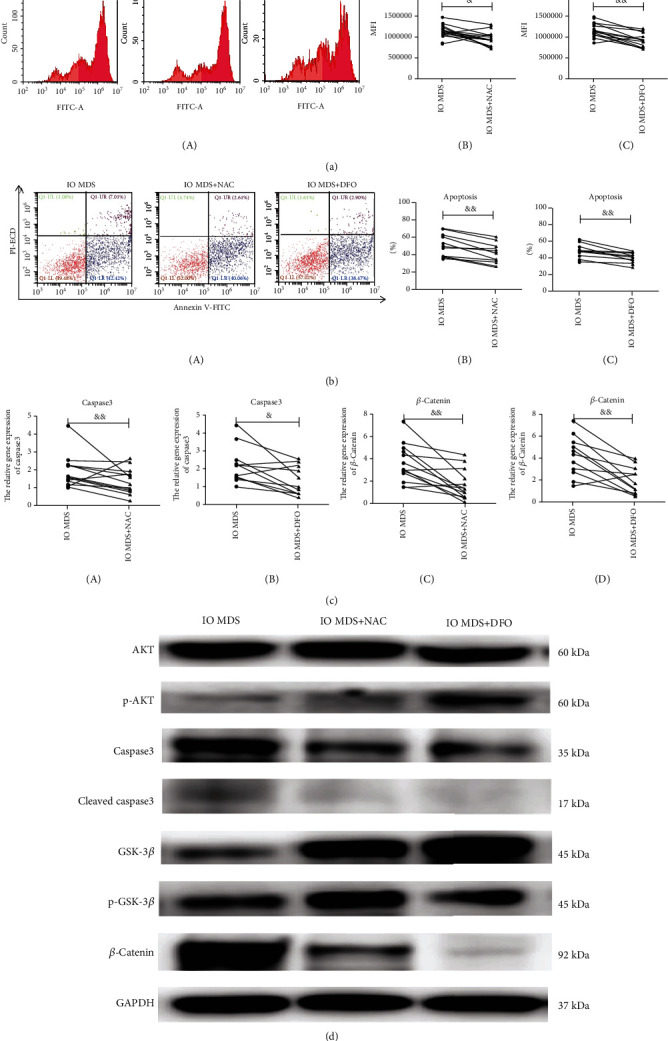
The effects of antioxidant or iron chelation treatments on IO MDS MSCs. (a) The levels of ROS in IO MDS MSCs were significantly decreased after the treatments with NAC or DFO. (b) The apoptosis of IO MDS MSCs was significantly decreased after the treatments with NAC or DFO. (c) The gene expressions of caspase3 and *β*-catenin in IO MDS MSCs were significantly downregulated after the treatments with NAC or DFO. (d) The protein of p-AKT in IO MDS MSCs was upregulated after the treatments with NAC or DFO, while caspase3 was downregulated. The protein of *β*-catenin in IO MDS MSCs was downregulated after the treatments with NAC or DFO, while GSK-3*β* was upregulated. Compared with the IO MDS group: & indicates *p* < 0.05; && indicates *p* < 0.01.

The cell proliferation ability was evaluated by the CCK8 assay. As shown in Figure 1(c), the proliferation ability of MSCs from MDS patients significantly decreased, and it significantly decreased in the IO MDS group compared with that in the NIO MDS group on days 6, 8, and 10 (*p* < 0.05) (Figure 1(c)). Meanwhile, proliferation-related protein p-AKT was downregulated in the IO MDS group (Figure 2(e)), which was upregulated after the treatments with NAC or DFO (Figure 3(d)). To confirm the effect of IO on the osteogenic differentiation ability of MSCs, cells were isolated and cultured in induction medium for 21 days. After 3 weeks of induction, the mineralized nodules staining with silver nitrate that formed in the IO MDS group were less than those in the NIO MDS group (Figure 1(d)).

### 3.4. IO Inhibited Expressions of VEGFA, CXCL12, and TGF-*β*1 Involved in Hematopoietic Regulation in MSCs

Meanwhile, the expressions of hematopoiesis chemokines in MSCs were also investigated. The gene expressions of VEGFA, CXCL12, and TGF-*β*1 regulating hematopoiesis were significantly downregulated in the IO MDS group compared with those in the NIO MDS and control groups (*p* < 0.05) (Figure 1(e) A–C). The level of TGF-*β*1 in the culture supernatants secreted by MSCs in the IO MDS group (1871.79 ± 601.87 pg/ml) was significantly lower than that in the NIO MDS group (2610.59 ± 464.51 pg/ml) and the control group (3323.03 ± 724.18 pg/ml) (*p* < 0.05) (Figure 1(e) D). The results suggested that IO might inhibit the hematopoiesis chemokines secreted by MSCs. Moreover, IO MDS MSCs treated with NAC or DFO showed significantly upregulated gene expressions of the three chemokines above (*p* < 0.05) (Figures 1(f)–1(h)), which indicated that damages of MSCs caused by IO could be partially alleviated by antioxidant or iron chelation treatments.

### 3.5. The Increased ROS Induced the Apoptosis of IO MDS MSCs

Due to the decreased quantity and abilities of MSCs, we further investigated the apoptotic effects of IO on MSCs. The apoptosis of the IO MDS group (47.49 ± 11.38%) was significantly higher than that in the NIO MDS group (38.16 ± 6.86%) and the control group (30.24 ± 8.51%) (*p* < 0.01) (Figure 2(a)). To clarify the role of ROS in apoptosis of MSCs, we examined the levels of ROS in MSCs. The level of ROS in the IO MDS group (1188503.71 ± 151641.12) was significantly higher than that in the NIO MDS group (1031773.06 ± 125481.41) and the control group (911600.09 ± 134124.83) (*p* < 0.05) (Figure 2(b)), which was parallel with apoptosis. Meanwhile, the gene expression of caspase3 was significantly upregulated (Figure 2(c)), with the increased protein levels of caspase3 and cleaved caspase3 (Figure 2(e)). Additionally, IO MDS MSCs treated with NAC or DFO showed significantly decreased levels of ROS and apoptosis (*p* < 0.05), as well as the apoptotic-related gene or protein caspase3 (Figures 3(a), 3(b), and 3(c) A–B), which indicated that the activated apoptosis signal pathway in IO MDS MSCs induced by increased ROS could be partially alleviated by antioxidant or iron chelation treatments.

### 3.6. The Activation of ROS-Related Wnt/*β*-Catenin Signaling Pathway in IO MDS MSCs

We further detected the gene and protein expressions involved in the ROS-related Wnt/*β*-catenin signaling pathway of MSCs, which might be involved in MDS progression. We found that the gene expression of *β*-catenin was significantly upregulated in the IO MDS group (Figure 2(d)), as well as the increased protein levels of *β*-catenin, while GSK-3*β* was decreased in the protein level (Figure 2(e)). To evaluate the effects of increased ROS in the regulation of the cell signaling pathway caused by IO, IO MDS MSCs were incubated with DFO or NAC, and the expressions of genes and proteins were further investigated. After the treatments with DFO or NAC, the gene and protein expression of *β*-catenin was downregulated in IO MDS MSCs, while GSK-3*β* was upregulated in the protein level (Figures 3(c) and 3(d)).

### 3.7. Comparisons between MSCs from Higher-Risk IO MDS or IO MDS/AML Patients

**Figure 4:**
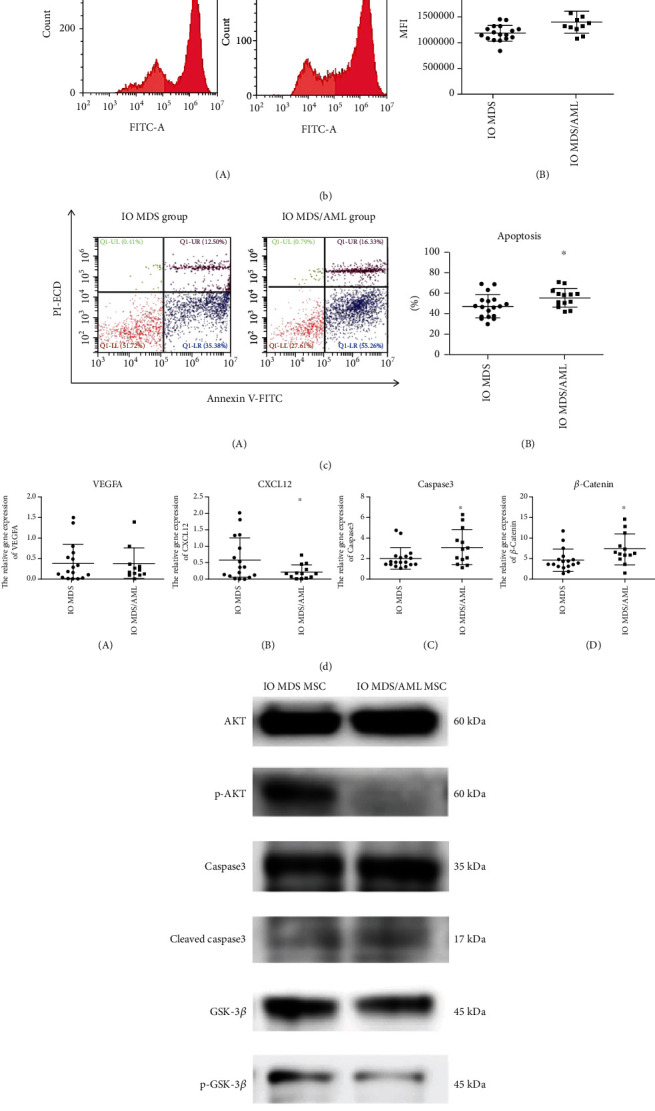
The characteristics and functions of MSCs between IO MDS and IO MDS/AML patients. (a) There was no difference in morphology of MSCs between IO MDS and MDS/AML patients. (b) The level of ROS in the IO MDS/AML group was significantly higher than that in the IO MDS group (*p* < 0.05). (c) The apoptosis of MSCs in the IO MDS/AML group was significantly higher than that in the IO MDS group. (d) The gene expressions of caspase3 and *β*-catenin in MSCs in the IO MDS/AML group were significantly upregulated compared with those in the IO MDS group, with a significantly downregulated gene expression of CXCL12. (e) The protein levels of p-AKT and GSK-3*β* in the IO MDS/AML group were downregulated, with upregulated protein levels of caspase3 and *β*-catenin. Compared with the IO MDS group: ∗ indicates *p* < 0.05.

Due to the activated ROS/Wnt signaling pathway in IO MSCs from higher-risk MDS patients, we further compared the differences in MSCs between IO MDS and IO MDS/AML patients. There was no obvious morphological difference between the IO MDS group and the IO MDS/AML group (Figure 4(a)). The level of ROS in the IO MDS/AML group (1399886.50 ± 215289.90) was significantly higher than that in the IO MDS group (1188503.71 ± 151641.12) (*p* < 0.05) (Figure 4(b)), with a significantly higher apoptosis rate of MSCs in the IO MDS/AML group (55.96 ± 9.28%) than that in the IO MDS group (47.49 ± 11.38%) (*p* < 0.05) (Figure 4(c)). Compared with the IO MDS group, the IO MDS/AML group had a decreased protein level of p-AKT (Figure 4(e)). Furthermore, the gene expressions of caspase3 and *β*-catenin in the IO MDS/AML group (3.08 ± 1.68; 7.25 ± 3.65) were significantly higher than that in the IO MDS group (1.99 ± 1.07; 4.64 ± 2.63) (*p* < 0.05), as well as the protein levels of caspase3 and *β*-catenin. The gene expression of CXCL12 (0.21 ± 0.22) in the IO MDS/AML group was significantly lower than that in the IO MDS group (0.59 ± 0.66) (*p* < 0.05). There was no obvious difference between the gene expression of VEGFA (0.36 ± 0.39) in the IO MDS/AML group and the IO MDS group (0.39 ± 0.46) (*p* > 0.05) (Figure 4(d)).

## 4. Discussions

MDS is common in the elderly, and the correlation between age of onset and MDS indicates that genetic damage is related to risk exposure or genetic susceptibility. One of the significant features of MDS is genomic instability, and most patients eventually progress to acute myeloid leukemia [17]. The majority of genetic abnormalities are caused by the loss or acquisition of large fragments of chromosomes, such as -7, 5q-, -5, and +8 [18]. Somatic point mutations are common in MDS patients and are associated with specific clinical characteristics; ASXL1, TP53, EZH2, ETV6, and RUNX1 gene mutations among them are independent predictors of poor overall survival in MDS patients [19]. Therefore, gene mutations caused by various reasons may be involved in the occurrence and development of MDS.

Iron overload is mostly caused by repeated chronic blood transfusions in MDS patients, while in some other patients, it is caused by myeloid dysplasia or increased intestinal iron absorption due to abnormal expression of hepcidin [6, 7]. Malcovati et al. showed that the transfusion-dependent MDS patients had a significantly shorter survival than that of non-transfusion-dependent patients, and the occurrence of secondary iron overload might significantly affect the survival of transfusion-dependent MDS patients [20]. Oxidative stress caused by IO induced DNA damage of peripheral blood cells in MDS patients, which was involved in the progression of disease [21]. However, DFX could reduce the DNA damage in cells of transfusion-dependent MDS patients caused by oxidative stress [22]. Iron chelation may not only improve the hematopoietic function of MDS patients, but also delay leukemia transformation. Iron chelators or strong antioxidants may play a positive role by reducing ROS levels [23]. Therefore, iron overload plays an important role in the occurrence and development of MDS.

MDS patients tend to have genetic instability, and iron overload is closely related to oxidative stress; excessive ROS may cause DNA damage, leading to mutation or genetic instability. Iron overload may activate protumor genes and inactivate tumor suppressor genes by inducing ROS, and finally, they may activate the signal pathway related to tumor generation. Meanwhile, ROS may promote tumor survival by mediating various epigenetic changes [24]. In our study, specific gene mutation screening was carried out in patients with intermediate or high-risk MDS. Further analysis showed that among the 34 gene mutations, IO MDS patients had a higher incidence of ASXL1 and TET2 gene mutations. Haferlach et al. showed that 47 genes were mutated in MDS patients, and more than 10% of them were mutated with TET2, SF3B1, ASXL1, SRSF2, DNMT3A, and RUNX1, which were mostly associated with the high risk of disease and the proliferation of blasts [25]. From clonal hematopoiesis to secondary leukemia of MDS, both TET2 and ASXL1 gene mutations are common in MDS and secondary leukemia patients. In MDS patients, TET2 gene mutation was associated with advanced age and normal chromosome karyotype [26], while in patients with AML, it indicated a poor prognosis [27]. ASXL1 gene mutation was associated with IPSS intermediate risk, increased blasts, short survival, and significantly shortened transformation time to leukemia [28]. Studies have shown that disturbing ROS homeostasis may affect the gene expressions of ASXL1 and TET2 [29, 30], but whether the increased ROS caused by iron overload may induce the gene mutations of ASXL1 or TET2 remains unknown and needs to be further studied. However, iron overload-related gene instability still plays an important role in the progression of MDS.

As an important part of the bone marrow microenvironment, MSC regulates hematopoiesis by secreting varieties of cytokines and affecting the self-renewal, proliferation, and differentiation of HSCs [31]. Previous studies showed that IO MDS patients had poor prognosis, and blood transfusion dependence often indicated the severity of diseases [32]. The quantity and functions of MSCs are abnormal in MDS, and IO may affect the quantity of MSCs and inhibit the hematopoiesis [3, 33]. In our study, according to the minimal criteria for defining human MSCs [34], MSCs were isolated and cultured in vitro, adherent cells were digested by trypsin and then passed on, and the third passage MSCs were used for subsequent study. The surface markers of MSCs were highly expressed CD73, CD90, and CD105, without expressed CD34 and CD45 of HSCs. The iron deposition in the IO MDS group was increased. The quantity of MSCs was reduced in the IO MDS group, without significant differences in morphology. Moreover, the proliferation ability of IO MDS MSCs was significantly decreased in the middle stage. We further examined the level of proliferation-related protein AKT. Results showed that the protein level of p-AKT was significantly decreased in the IO MDS group, which could be partly reversed by antioxidant or iron chelation treatments. By regulating the expressions of GSK-3, mTORC1, and other key downstream signals, AKT is involved in multiple physiological processes such as cell survival, proliferation, and metabolism [35]. Meanwhile, the osteogenic differentiation ability of IO MDS MSCs was decreased, which might be related to decreased hematopoietic functions. It was consistent with the previous study about IO decreasing the osteogenic differentiation ability of MSCs [36]. Our results show that IO can induce the decreased number and osteogenic differentiation ability of MSCs from MDS patients, and impair the proliferation ability of MSCs by affecting the AkT-related signaling pathway.

MSCs regulate and support hematopoiesis by secreting varieties of cytokines [37]. Abbas et al. suggested that HSC maintenance genes such as CXCL12 and VEGFA in MSCs were downregulated in MDS patients [38]. Our results showed that IO inhibits the gene expressions of VEGFA and CXCL12 in higher-risk MDS patients. After the treatments with antioxidant or iron chelation, the two gene expressions above in IO MDS MSCs were upregulated, which was conducive to the hematopoiesis of MDS patients. The decreased quantity and proliferation ability of IO MSCs may account for the decreased expressions of VEGFA and CXCL12, and further affect the bone marrow hematopoiesis. The TGF-*β* signaling pathway was involved in cell proliferation, differentiation, and apoptosis, and played inhibitory roles in hematopoiesis regulation and tumor progression [39]. Meanwhile, TGF-*β* was important in maintaining the self-renewal and survival of HSCs. Our results showed that IO inhibits both gene expression and secretion of TGF-*β*1 in higher-risk MDS patients. The decreased quantity and reduced TGF-*β*1 expression of IO MDS MSCs might weaken the inhibitory effects to MDS malignant clones. Although TGF-*β*1 could inhibit normal hematopoiesis, its inhibitory effects on malignant cells were more obvious, which might lead to the progression of MDS.

The decreased quantity and functions of MSCs caused by IO affected the hematopoiesis of MDS patients. A variety of mechanisms were involved in IO damaging MSCs. ROS played an important role in IO-related injury. Mitochondria was the main organelle producing ROS, which was also the organelle damaged by ROS in turn. The apoptosis of IO MDS MSCs was induced by increased ROS, which activated the intracellular AMP-activated protein kinase (AMPK)/mitochondrial fission factor (MFF)/mitochondrial fission protein 1 (Drp1) signaling pathway [13]. The damages of MSCs caused by IO could be partially reversed by reducing ROS and inhibiting p53, ERK, p38, and other signaling pathways [40, 41]. We found that the increased level of ROS in MSCs caused by IO might be inducing apoptosis, while the treatments with antioxidant or iron chelation could significantly decrease the levels of ROS and apoptosis in IO MSCs. Furthermore, the gene and protein levels of caspase3 in IO MDS patients were significantly upregulated, which could be partially reversed by an antioxidant or iron chelator. It indicated that IO could induce the apoptosis of MSCs by activating the ROS-related apoptosis signaling pathway.

The Wnt/*β*-catenin signaling pathway was important in cell proliferation, survival, and tumor progression. In patients with acute T-lymphocytic leukemia, *β*-catenin was highly expressed in leukemia stem cells, while the inactivation of *β*-catenin significantly reduced the quantity of leukemia stem cells, indicating that the activation of *β*-catenin signals might be involved in survival and proliferation of leukemia stem cells [42]. The expression of *β*-catenin in MSCs of mouse with MDS was abnormal, and gene knockout of *β*-catenin could inhibit the progression of MDS in mice and prolong the survival time, indicating that the abnormal activation of *β*-catenin in MSCs was related to the progression of MDS [43]. Our results showed that both the gene and protein expressions of *β*-catenin were significantly increased in the IO MDS group, while protein p-GSK-3*β* significantly decreased. In the classical Wnt signaling pathway, p-GSK-3*β* was essential in the phosphorylation and proteasomal degradation of *β*-catenin, and it inhibited the *β*-catenin signal pathway [44]. Previous studies have shown that deferasirox (DFX) could inhibit the proliferation of MM cells, reduce intracellular ROS, and inhibit the expressions of proline-rich tyrosine kinase 2 (Pyk2) and the downstream Wnt/*β*-catenin signal pathway [45]. After the treatments with antioxidant or iron chelation, the levels of ROS in the IO MDS group significantly decreased, with the downregulated *β*-catenin in gene and protein levels and the upregulated protein p-GSK-3*β*. It is suggested that IO might increase the level of ROS, inhibit the protein p-GSK-3*β*, and reduce the degradation of *β*-catenin. Finally, the expression of intracellular *β*-catenin was upregulated, which might be involved in the progression of MDS.

From the study above, we found that IO impairs the MSCs from higher-risk MDS patients by activating the ROS-related Wnt signaling pathway. Therefore, we further compared the differences between MSCs in IO MDS/AML and IO higher-risk MDS patients. We found that the IO MDS/AML group had a decreased quantity of MSCs compared with the IO MDS group without morphological differences. The gene expression of CXCL12 was significantly decreased in the IO MDS/AML group. The reduced number and function of MSCs in the MDS/AML group prompted us to explore its mechanism. We found that the expression of proliferation-related protein p-AKT was significantly downregulated. Meanwhile, the levels of ROS and apoptosis of MSCs in IO MDS/AML group were significantly increased compared to those in the IO MDS group, with significantly increased expressions of caspase3 and *β*-catenin. Previous studies showed that abnormal activation of the Wnt/*β*-catenin signaling pathway in leukemia stem cells (LSCs) was related to the proliferation of LSCs, and which in MSCs of MDS was associated with the progression of MDS [42, 43]. In our study, the gene and protein expressions of *β*-catenin were significantly upregulated in IO MDS patients, especially in IO MDS/AML patients. We speculate that IO may exert an inhibitory effect on MSCs by the decreased quantity and functions of MSCs in MDS/AML patients. Meanwhile, the increased level of ROS might be activating the Wnt/*β*-catenin signal pathway in IO MSCs and promoting the progression of MDS.

In conclusion, IO decreased the quantity and weakened the proliferation and osteogenic differentiation abilities of MSCs, as well as inhibited the gene expressions of VEGFA, CXCL12, and TGF-*β*1 related to hematopoiesis in higher-risk MDS patients. The increased levels of ROS caused by IO might be inducing apoptosis in MDS MSCs by activating the caspase3-related apoptosis signaling pathway. Meanwhile, the activated Wnt/*β*-catenin signal pathway in IO MDS and IO MDS/AML MSCs may participate in the progression of MDS. Both antioxidant or iron chelation treatments could partly alleviate the damages of MSCs caused by IO in MDS patients.

## Figures and Tables

**Table 1:** Characteristics of patients.

	All patients	MDS patients		IO MDS/AML patients
		IO group	NIO group	
*N* (%)	53	18 (34.0%)	22 (41.5%)	13 (24.5%)
Median age (years)	61 (26 ~ 85)	56 (26 ~ 84)	57.5 (33 ~ 82)	64 (53 ~ 85)
Gender				
Male	23	9	7	7
Female	30	9	15	6
Ferritin (ng/ml)	1119.2 (32.72 ~ >2000)	2000 (1012.85 ~ >2000)	427.28 (32.72 ~ 874.57)	1831.1 (1040.8 ~ >2000)
2016 WHO classification				
SLD	1	0	1	
RS	2	1	1	
MLD	10	6	4	
EB-1	10	3	7	
EB-2	17	8	9	
IPSS-R				
Mediate risk	17	8	9	
High risk	13	4	9	
Very high risk	10	6	4	

No significant difference was found in the distribution of gender, WHO classification, and IPSS-R between the NIO MDS group and the IO MDS group (*p* > 0.05). IO: iron overload; NIO: noniron overload; MDS/AML: MDS transformation to AML.

**Table 2:** Gene-specific primers used in RT-PCR.

Genes	Primer sequence (5′–3′)
VEGFA	Forward 5′AGCCTTGCCGCCTTGCTGCTCTA3′ Reverse 5′GTGCTGGCCTTGGTGAGG3′
CXCL12	Forward 5′GAGCTACAGATGCCCATGC3′ Reverse 5′CTTTAGCTTCGGGTCAATGC3′
TGF-*β*1	Forward 5′GCGTGCTAATGGTGGAAACC3′ Reverse 5′GCTTCTCGGAGCTCTGATGTG3′
Caspase3	Forward 5′TTGTAGAAGTCTAACTGGAA3′ Reverse 5′CCATGTCATCATCAACAC3′
*β*-Catenin	Forward 5′GCAGCTGCTGTTTTGTTCCG3′ Reverse 5′GATTGCTGTCACCTGGAGGC3′
*β*-Actin	Forward 5′TGGACATCCGCAAAGACCTGT3′ Reverse 5′CACACGGAGTACTTGCGCTCA3′

**Table 3:** Next generation sequencing of the intermediate or high-risk MDS patients.

	NIO MDS group (11)	IO MDS group (10)	*p* value
Presence of gene mutation	8 (72.7%)	10 (100%)	0.214
STAG2	1 (9.1%)	0 (0)	1.000
BCOR	1 (9.1%)	0 (0)	1.000
ASXL1	1 (9.1%)	6 (60.0%)	**0.024**
U2AF1	1 (9.1%)	3 (30.0%)	0.311
RUNX1	3 (27.3%)	1 (10.0%)	0.586
ZRSR2	1 (9.1%)	0 (0)	1.000
WT1	1 (9.1%)	2 (20.0%)	0.586
GATA2	1 (9.1%)	3 (30.0%)	0.311
TET2	2 (18.2%)	7 (70.0%)	**0.030**
TP53	1 (9.1%)	0 (0)	1.000
NRAS	1 (9.1%)	0 (0)	1.000
SETBP1	1 (9.1%)	1 (10.0%)	1.000
DNMT3A	1 (9.1%)	0 (0)	1.000
IDH	0 (0)	1 (10.0%)	0.476
EZH2	0 (0)	1 (10.0%)	0.476

## Data Availability

The datasets used and/or analyzed during the current study are available from the corresponding author on reasonable request.

## Abbreviations

AML: Acute myeloid leukemia
BMMNC: Bone marrow mononuclear cells
DCFH-DA: 2′,7′-Dichlorofluorescein diacetate
DFO: Deferoxamine
IO: Iron overload
LSCs: Leukemia stem cells
MDS: Myelodysplastic syndrome
MSCs: Mesenchymal stromal cells
NAC: N-Acetylcysteine
NIO: Noniron overload
ROS: Reactive oxygen species.

## Supplementary Materials

Supplementary Materials Table S1: the list of gene mutations detected in MDS patients.
