# Supplementary material for: Iron Overload Impairs Bone Marrow Mesenchymal Stromal Cells from Higher-Risk MDS Patients by Regulating the ROS-Related Wnt/β-Catenin Pathway
Source: Stem Cells Int. 2020 Oct 31;2020:8855038. doi: 10.1155/2020/8855038 (PMC7648692; doi:10.1155/2020/8855038)
Supplement: Supplementary Materials — Table S1: the list of gene mutations detected in MDS patients. [file 8855038.f1.docx]

TableS1 The list of gene mutations detected in MDS patients

| number | Gene | number | Gene | number | Gene | number | Gene |
| --- | --- | --- | --- | --- | --- | --- | --- |
| 1 | ASXL1 | 10 | IDH1 | 19 | SF3B1 | 28 | KIT |
| 2 | BCOR | 11 | IDH2 | 20 | SH2B3 | 29 | NPM1 |
| 3 | BCORL1 | 12 | JAK2 | 21 | SRSF2 | 30 | GATA2 |
| 4 | CARL | 13 | KRAS | 22 | TET2 | 31 | MLL |
| 5 | CBL | 14 | MPL | 23 | TP53 | 32 | PDGFRA |
| 6 | CSF3R | 15 | NRAS | 24 | U2AF1 | 33 | PHF6 |
| 7 | DNMT3A | 16 | PIGA | 25 | ZRSR2 | 34 | WT1 |
| 8 | ETV6 | 17 | RUNX1 | 26 | CEBPA |  |  |
| 9 | EZH2 | 18 | SETBP1 | 27 | FLT3 |  |  |
